# What are the dominant cytokines in early rheumatoid arthritis?

**DOI:** 10.1097/BOR.0000000000000470

**Published:** 2017-12-04

**Authors:** Laura A. Ridgley, Amy E. Anderson, Arthur G. Pratt

**Affiliations:** National Institute for Health Research Newcastle Biomedical Research Centre, Newcastle upon Tyne Hospitals NHS Foundation Trust and Newcastle University, Newcastle upon, Tyne, UK

**Keywords:** chemokine, cytokines, interleukin, pathogenesis, rheumatoid arthritis

## Abstract

**Purpose of review:**

Rheumatoid arthritis is a systemic disease of evolving immune dysregulation that culminates in joint destruction and disability. The principle by which pro-inflammatory cytokines may be therapeutically targeted to abrogate disease is well established, but has yet to translate into reliable cures for patients. Emerging insights into cytokine-mediated pathobiology during rheumatoid arthritis development are reviewed, and their implications for future treatment strategies considered.

**Recent findings:**

Accumulating data highlight cytokine perturbations before the clinical onset of rheumatoid arthritis. Some of these have now been linked to the arthritogenic activation of autoantibodies and associated pain and bone destruction in affected joints. These observations suggest cytokines may trigger the transition from systemic immunity to arthritis. Cytokine exposure could furthermore ‘prime’ synovial stromal cells to perpetuate a dominant pro-inflammatory environment. By facilitating cross-talk between infiltrating immune cells and even sustaining ectopic lymphoid structure development in some cases, cytokine interplay ultimately underpins the failure of arthritis to resolve.

**Summary:**

Successful therapeutic stratification will depend upon an increasingly sophisticated appreciation of how dominant players amongst cytokine networks vary across time and anatomical space during incipient rheumatoid arthritis. The prize of sustained remission for all patients justifies the considerable effort required to achieve this understanding.

## INTRODUCTION

Rheumatoid arthritis is a systemic inflammatory disease that primarily affects the synovial joints, and for which there is no known cure. Its heterogeneity, instantly recognizable to clinicians in the variability of its clinical presentation, is multilayered, confounding a unified description of pathogenesis. In particular, the concept that distinct subtypes of the syndrome are delineated by the presence or absence of circulating antibodies to citrullinated peptides (ACPAs) has gained traction recently [1,2]. In ACPA ‘seropositive’ disease, elegant epidemiological and translational work converges on a stepwise model for disease development [1] in which cigarette smoke exposure, other environmental effects and the microbiome act as principal risk factors for autoantibody development long before symptom onset [3–5]. Genetically determined amino acid sequences in the MHC binding groove of antigen presenting cells then gain influence in driving accelerated autoimmunity and the transition to arthritis in at-risk individuals [1,6]. Mechanisms behind the development of seronegative rheumatoid arthritis remain far less well understood, its heritability and association with smoking both apparently modest by comparison, but a recent familial aggregation study suggests the aetiological overlap between these serotypes may be more important than yet fully appreciated [7]. Running through this complex backdrop of disease initiation, and orchestrating the common phenotype of persistent synovial hyperplasia, immune cell infiltration and joint destruction that ensues, the fundamental importance of cytokines in rheumatoid arthritis pathogenesis is long established (Table 1).

**FIGURE 1:**
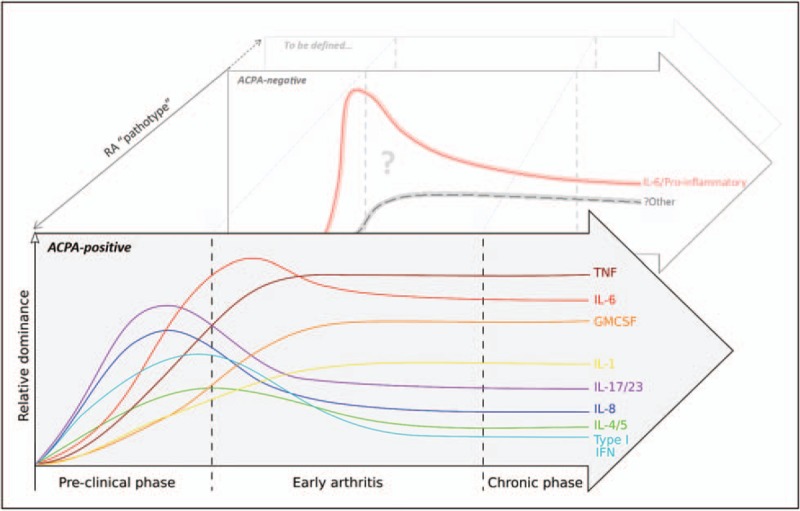
The hierarchical dominance of cytokines during rheumatoid arthritis development is dynamic. Recent insights permit a speculative depiction of how individual cytokines may exhibit distinct and evolving patterns of relative importance, even during the earliest phases of ACPA-seropositive rheumatoid arthritis. Whilst pro-inflammatory cytokines are clearly important drivers of seronegative disease, a paucity of equivalent data for this subset currently precludes a similarly granular representation of this or other, as yet undefined, rheumatoid arthritis ‘pathotypes’.

Cytokines are typically secreted by leukocytes to exert paracrine or autocrine effects, thereby regulating such diverse functions as cellular differentiation, activation, migration and survival. Exhibiting pleiotropy individually and synergy or redundancy in concert, the sensitivity of their combinatorial networks to perturbation is exquisite, and their individual potential for exploitation as therapeutic targets well recognized. Blockade of signalling by pro-inflammatory cytokines, such as tumour necrosis factor (TNF) and interleukin-6 (IL-6), using monoclonal antibodies has revolutionized outcomes for patients with severe rheumatoid arthritis but, in common with all currently available biologics, these drugs remain subject to a ‘therapeutic ceiling’, with true remission unattainable for the majority. Replicating even this degree of success in the clinic has proved difficult in clinical trials of alternative agents, for example blocking IL-1β, IL-17 and the IL-12/23 family in rheumatoid arthritis – despite their attractiveness as targets [8–10]. Rather than being interpreted as evidence that these cytokines are pathogenetically unimportant, such setbacks should prompt a more nuanced critique of our current therapeutic approach. An increasingly sophisticated appreciation of how ‘dominant’ players within cytokine hierarchies may vary over time, across tissue boundaries and between individuals during incipient rheumatoid arthritis is now emerging (Fig. 1). Developing and harnessing this understanding, perhaps guided by judicious sampling of blood or tissue at an individual patient level to more rationally map therapeutic strategies to disease endotypes, should one day pay dividends in the clinic. The present review will show how recent insights into the role of cytokines in the earliest stages of rheumatoid arthritis have advanced this endeavour. 

**Box 1:**
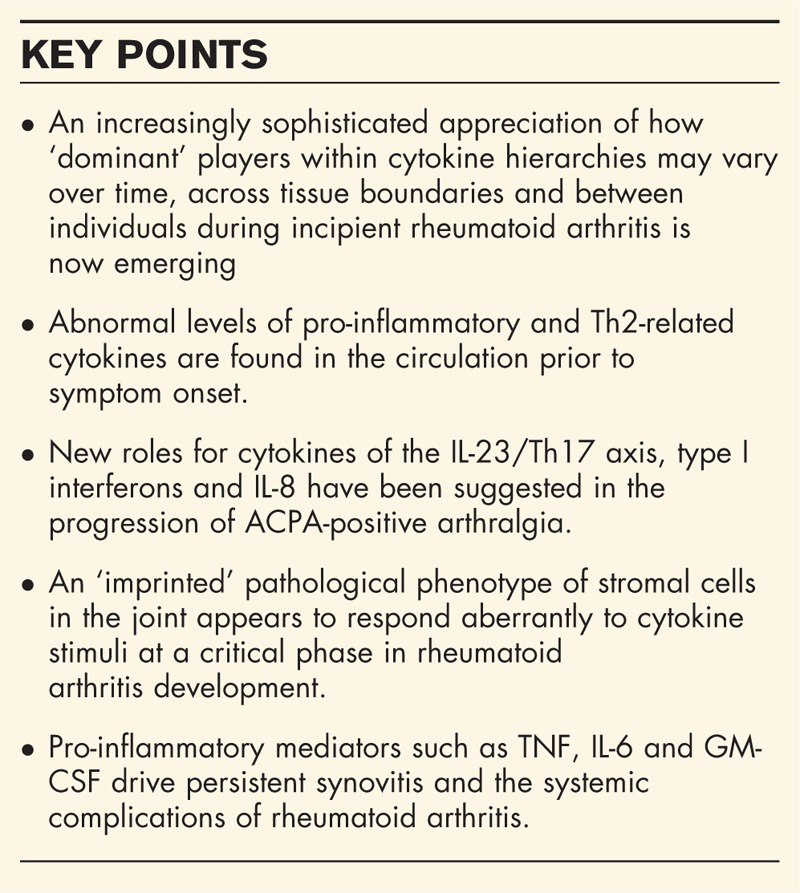
no caption available

## CYTOKINES AS COORDINATORS OF PRE-CLINICAL RHEUMATOID ARTHRITIS

Genes encoding functional components of the immune system are enriched within loci associated with the development and natural history of rheumatoid arthritis; protein products pathogenetically implicated include cytokines themselves [IL-2, IL-21, G-CSF and granulocyte macrophage-colony stimulating factor (GM-CSF)], their receptor components [for IL-6, IL-20, interferon (IFN)-γ and IL-2] and elements of their downstream signalling machinery (e.g. TYK2, STAT4 and TNFAIP3). The range of epigenetic and other mechanisms via which such variants might disrupt cytokine homeostasis to confer disease risk is only beginning to be understood, together with an appreciation that they will best be dissected at a cellular level in relevant disease contexts [11,12]. This becomes pertinent when considering measurable alterations in a number of circulating cytokines that have, with some consistency, been observed prior to symptom onset amongst those who subsequently develop rheumatoid arthritis compared with healthy individuals. These include increased pro-inflammatory examples (TNF, IL-6, IL-1β and/or IL-1RA, GM-CSF) as well as IL-4, IL-12, IL-17 and the eosinophil chemotactic chemokine, eotaxin (Table 1). Against a facultative genetic background, such mediators may variously arise from the paucicellular joint itself, bone marrow or elsewhere in the periphery, but their presence reinforces the likely contribution of cytokines to systemic and general immune dysregulation prior to overt synovitis. In a recent study interrogating serum analyte profiles of ACPA seropositive patients with joint pain (arthralgia), the discriminatory value of the Th2-specific cytokine IL-5 for rheumatoid arthritis progression was highlighted [13^▪^]. Together with the elevated circulating IL-4 and eotaxin mentioned above, these data recall much earlier observations of transient Th2 cytokine profiles in synovial fluid of early seropositive synovitis patients who progressed to rheumatoid arthritis [14]. Th2 effector responses protect against inflammatory arthritis in certain contexts [15], and the extent to which such observations reflect ‘failed regulation’ or merely emphasize the role of humoral responses during preclinical rheumatoid arthritis remains to be clarified (Table 1).

The functional diversity of cytokines found in the circulation of individuals at risk of rheumatoid arthritis may be simplest to rationalize by viewing serum merely as a conduit between distant tissue sites, each harbouring an immunologically discrete niche; in acknowledgment of this it was proposed that measuring circulating mediators *en masse* to derive a summary ‘cytokine score’ for disease prediction might be their most practical application [16]. Nonetheless, examples of how specific cytokines actively shape antibody-mediated autoimmunity in the run-up to clinically manifest rheumatoid arthritis are now emerging. In the case of the IL-23-Th17 axis their propensity to do so appears greater during this ‘prearticular’ phase than after synovitis has developed (Fig. 1). Hence, recent data from mouse models of autoimmune arthritis show that IL-23-activated Th17 cells can, via IL-21 and IL-22, ‘programme’ germinal centre plasmablasts and plasma cells to alter the Fc glycosylation profile of secreted IgG autoantibodies [17^▪▪^]. Reduced terminal glycan sialylation at the asparagine-297 position thereby augments autoantibody affinity for osteoclast Fc receptors, impacting their propensity to localize to the joint and mediate bone loss [18]. Critically, these changes in autoantibody glycosylation directly mirror those seen to occur in circulating ACPAs of seropositive patients as their arthritis develops [17^▪▪^]. Recent experiments also support a nonredundant role for IL-8 in ACPA-mediated osteoclast activation as a mechanism of bone loss, and even arthralgia, prior to arthritis development [19^▪▪^,20^▪^]. Here it was shown that peptidyl arginine deiminase enzymes necessary for osteoclast differentiation had, as a result of their citrullinating activity, a secondary effect of generating additional targets for circulating ACPA within the joint; IL-8 was specifically produced by these cells in response to ACPA, feeding an autocrine loop that resulted in bone loss [19^▪▪^] and (in mice) pain-like behaviour [20^▪^]. Considered together these data are exciting as they point to a hitherto elusive model to explain how systemic autoimmune propensity manifests as joint-specific diseases; its confirmation is now eagerly awaited.

Type I interferons including the prototype IFNα, produced mainly by plasmacytoid dendritic cells, may provide a further example of cytokine-mediated autoantibody modulation. These factors are known to promote a number of functions linked to autoimmune pathology including B-cell differentiation and IgG class-switching. Up-regulation of their target gene expression in whole blood (interferon gene signature, IGS), used as a surrogate of circulating cytokine levels, has been shown to predict IgG development in at-risk, ACPA+ individuals, as reflected predominately by its presence in polymorphonuclear granulocytes [21^▪▪^], and IGS elements were recently shown to correlate with ACPA titres in a Mexican population [22].

Studying the preclinical phase of seronegative rheumatoid arthritis poses a unique and important challenge now beginning to be addressed [23^▪^]. Of interest, patients with this subgroup of disease typically describe a shorter symptom duration when they present with arthritis, but with evidence for more prominent IL-6-mediated lymphocyte activation in the periphery, compared with their seropositive counterparts [24,25], consistent with a more ‘explosive’, pro-inflammatory component to the natural history of seronegative disease (Fig. 1). The knowledge that adaptive immune activation may be facilitated by pro-inflammatory cytokines in the absence of ongoing antigenic stimulus [26] suggests alternative mechanisms of sustained immune dysregulation that warrant further study in this subgroup.

## CYTOKINES IN THE TRANSITION TO CHRONICITY

The pathological hallmarks of synovitis in rheumatoid arthritis include the proliferation of resident synovial fibroblasts, new blood vessel formation and the recruitment of a wide range of leukocytes including B and T lymphocytes, monocytes/macrophages and mast cells; in turn this leads to synovial hypertrophy and the invasion of cartilage and bone by activated inflammatory tissue. Cytokines are fundamental orchestrators of the development and maintenance of this lesion. Amongst them, TNF appears to hold a position of hierarchical dominance during the inflammatory disease phase, promoting the activation of osteoclasts, chondrocytes, vascular endothelium and fibroblasts, and so directly mediating synovial hypertrophy and damage whilst in turn up-regulating the expression of other locally abundant pro-inflammatory cytokines. These include members of the IL-1 family, IL-6 and GM-CSF, the latter two of which clearly possess nonredundant functions, respectively in T-cell activation and the differentiation of inflammatory macrophages and dendritic cells. Furthermore, IL-6 and TNF exert potent systemic effects that help drive some of the co-morbidities seen in rheumatoid arthritis, including altered cholesterol metabolism, atherosclerosis and even mood disturbance [27^▪^]. Finally, in relation to adaptive immune dysregulation, these two cytokines were recently shown to induce the secretion of soluble programmed cell death-1 (sPD-1) by CD4^+^ T cells, competitively compromising the normal PD-1-mediated regulation of these cells’ activation in inflammatory arthritis patients [28^▪^].

The broad inflammatory features of rheumatoid synovitis are well described [27^▪^], but an emergent literature has now set ‘early synovitis’ apart as a distinct, transitional pathological phase in rheumatoid arthritis development, during which cytokine cross-talk between cells of the stroma, endothelium and the immune system may uniquely effect the failure of inflammation to resolve within the joint. Central to this concept is an appreciation that synovial fibroblasts, far from functioning merely as inert ‘joint scaffolding’, instead actively direct cellular interactions according to an epigenetically imprinted phenotype that is potentially more vulnerable to the cumulative effects of inflammation than stromal cells located elsewhere [29]. Building on this insight, Filer *et al.* recently demonstrated that an immune-protective effect exerted by TNF-exposed synovial fibroblasts from recent-onset arthritis patients in whom synovitis spontaneously resolves – whereby lymphocyte adhesion to endothelial cells in co-culture is prevented – was lost amongst synovial fibroblasts derived instead from patients with recent-onset rheumatoid arthritis, in whom synovitis persists [30^▪▪^]. The phenomenon appeared to be partly mediated by IL-6 which, although abundant in both ‘resolving’ and ‘persistent’ synovitis, mediated divergent effects during this circumscribed disease phase. On the other hand, only synovial fibroblasts from patients with advanced rheumatoid arthritis promoted lymphocyte adhesion even in the absence of TNF [30^▪▪^]. These data illustrate the importance of considering cytokine effects in the context of disease phase and diagnostic category, and may have consequences for the optimal therapeutic timing of cytokine blockade (Fig. 1). As with the preclinical disease phase, the autoantibody status should also be considered. For example, in patients with untreated early rheumatoid arthritis high circulating levels of IL-20 and IL-24 discriminate seropositive individuals and predict bony erosion – a property lost after treatment initiation; these cytokines appear to be produced by monocytes activated by immune complexes, and in turn mediate osteoclast activation [31^▪^].

Aside from the largely pro-inflammatory moieties discussed above, recent investigations into a number of ‘regulatory’ cytokines that shed light on means by which immune homeostasis might be restored during rheumatoid arthritis development are also considered. For example, the observation that spontaneous resolution of synovitis in a mouse model is dependent on IL-9 raised the intriguing possibility that this cytokine could also regulate human RA persistence: an enrichment of IL-9-producing type 2 innate lymphoid cells (ILC-2s) was indeed present in the circulation and synovium of patients with active rheumatoid arthritis compared with those on effective treatment and controls [32^▪▪^]. Tertiary lymphoid organs (TLOs) that closely resemble lymphoid follicles (with segregated T- and B-cell zones, follicular dendritic cell networks and a supporting stroma) are observed in approximately 40% of patient with rheumatoid arthritis synovium, where they support local autoantibody responses and may be a marker of adverse prognosis. It was recently shown that IL-27 inhibits TLO development via the inhibition of podoplanin-expressing Th17 cells [33]. Any therapeutic gains in the light of these insights remain some way off, but they offer a rich field for future research.

## CYTOKINE-TARGETED THERAPY: LESSONS AND FUTURE DIRECTIONS

Experience gained from the use of targeted therapies may teach us much about rheumatoid arthritis pathogenesis – including the hierarchical dominance of individual cytokines at its various phases. The value of achieving rapid and broad suppression of pro-inflammatory pathways during the earliest phase of arthritis by blocking TNF or IL-6-mediated signalling has been demonstrated, for example leading to sustained remission in about a third of patients [34,35]. Amongst patients with rheumatoid arthritis who fail to respond to ‘traditional’ nonbiological DMARDs these pathways clearly remain important drivers of disease for many, and GM-CSF blockade has now joined the above strategies as a rapidly effective treatment modality in this setting [36]. The efficacy of small molecular inhibitors of these cytokines’ downstream signalling machinery (the Janus kinase family) further supports their importance in perpetuating the inflammatory joint disease. By contrast, unsuccessful therapeutic targeting of other pro-inflammatory cytokines, despite their evident participation in pathogenesis, likely reflects functional redundancy during the disease phase at which clinical trials have thus far been undertaken. In the case of IL-1 this is true in both early and established rheumatoid arthritis, but attempts to block the IL-23/17 axis have thus far tended to be limited to patient groups with established disease [8,9]. Conceivably, based on recent insights described above, inhibitors of IL-23, p40 (the common subunit of IL-23 and IL-12) and IL-17A, may be more rationally deployed in seropositive individuals before clinically overt arthritis develops – in which context the role of Th17 cells could be more pivotal [17^▪▪^]. Here, the potential role of agents that block IL-8 signalling (such as the CXCR1/2-inhibitor reparixin) or IFN-α (e.g. sifalimumab) has become the subject of similar conjecture [37].

The preponderance of ACPA and/or rheumatoid factor seropositive patients with rheumatoid arthritis amongst populations studied in most of the clinical trials alluded to here is notable. Where it is available, accumulating data identifies seronegative rheumatoid arthritis as subject to relatively unfavourable treatment responses, irrespective of the targeted cytokine. Perhaps this is unsurprising given the aetiological distinction of this subgroup, but it highlights persistent unmet needs: not only in pathophysiological understanding, but also in our ability to map therapeutic response to measurable markers of the heterogeneous disease process at an individual patient level. Pretreatment levels of circulating cytokines themselves have so far proved to be of little value for this purpose, and the ability to measure their effects at a cellular level and/or within synovial tissue holds promise for stratified treatment approaches [38^▪^].

## CONCLUSION

Cytokines leave their footprint at all stages of the natural history of rheumatoid arthritis. Their signalling pathways are represented amongst genes encoded at disease risk loci; they regulate the immunological ‘prodrome’ that precedes clinically manifest arthritis – including autoantibody pathogenicity and joint pain; they mediate (and are mediated by) stromal dysregulation within the joint at the earliest stages of synovitis; and they define and perpetuate the chronic inflammation that ensues. Harnessing an understanding of how members of this diverse family of molecules variously dominate at each disease phase across disease subgroups should yield more rational and effective treatment strategies.

## Acknowledgements

Ms Ridgley's work is supported by the Arthritis Research UK Centre of Excellence for the Pathogenesis of Rheumatoid Arthritis (RACE); all authors gratefully acknowledge constructive discussions with colleagues and collaborators within this Centre that have informed the content of this review. The authors have benefitted from infrastructural support from the National Institute of Health Research (NIHR) Newcastle Biomedical Research Centre.

### Financial support and sponsorship

None.

### Conflicts of interest

Dr Pratt has received funding from Pfizer towards an externally peer-reviewed research project. Honoraria he has received from Eli Lilly and Janssen-Cilag Ltd. for his time in preparing presentations for nonpromotional meetings have been paid directly to Newcastle University. The views expressed in this manuscript are his own.

## REFERENCES AND RECOMMENDED READING

Papers of particular interest, published within the annual period of review, have been highlighted as:

▪ of special interest
▪▪ of outstanding interest

## Figures and Tables

**Table 1:** Overview of cytokines with established and emerging roles in early rheumatoid arthritis pathogenesis

Cytokine	Typical cellular source(s)	Cellular target(s) [and effect(s)]	Proposed role(s) in rheumatoid arthritis	Targeting strategy/ies
TNF	Monocytes/macrophages.	SFs (pro-inflammatory cytokine production)Osteoclasts (differentiation, activation)Endothelium (neovascularization)- Lymphocytes (Treg inhibition)	Pro-inflammatoryBone erosionSystemic (?fatigue)	Anti-TNF (infliximab^a^, adalimumab^a^, golimumab^a^, cerolizumab^a^), TNFR (etanercept^a^).
IL-6	Monocytes/macrophages, stroma/SFs	SFs (activation, proliferation)Macrophage (osteoclast differentiation)T cells (proliferation, survival, Th17 differentiation)B cells (survival, antibody production).Hepatocytes (acute phase reactants)	Pro-inflammatorySystemic (atherosclerosis, impaired lipid metabolism, anaemia)	Anti-IL-6R (tocilizumab^a^, sarilumab), anti-IL-6 (sirukumab)
IL-1α/β	Monocytes/macrophages, DCs	Osteoclasts (activation)T cells (Th17 differentiation)Endothelium (vasodilation)Autocrine (pro-inflammatory)	Pro-inflammatory (contributory rather than dominant role)	IL-1RA (anakinra), anti-IL-1β (canakinumab).
IL-17A/F	Th17 cells, neutrophils, ILCs, iNKT cells.	SFs (proliferation, pro-inflammatory cytokine production, including IL-6)- Chondrocytes (metalloproteinase induction)Myeloid cells/neutrophils (chemotaxis)Endothelium (neovascularization)	Pro-inflammatory (?contributory *versus* dominant role depending on disease subset)	Anti-IL-17A (secukinumab), anti-IL-17RA (brodalumab).
IL-23	Macrophages, DCs	Th17 cells (development, maintenance and expansion; IL-21/IL-22 induction)	Th17 responses	Anti-p40 (common subunit of IL-23/12; ustekinumab), anti-IL-23 (guselkumab)
IL-21	Th17 cells, Th2 cells, NK cells, Tfh cells.	B-cell maturation, plasma cell development/antibody production	?Role in arthritogenic autoantibody glycosylation (Ref [16])	Anti-IL-21 in development
IL-12	Macrophages, DCs	Th1 cells (differentiation, autocrine)	?Cell-mediated immune resposes, Th17 plasticity.	Anti-p40 (common subunit of IL-23/12; ustekinumab)
GM-CSF	Monocytes/macrophages, lymphocytes, stroma/SFs	Myeloid cells (differentiation/proliferation)Macrophages (pro-inflammatory phenotype)DCs (activation)	Pro-inflammatory, ?Pain	Anti-GM-CSF-Rα (mavrilimumab)
Th2 cytokines	Th2 cells, mast cells	Various	Awaits clarification [13^▪^,^14^,^15^]	Strategy to be determined.
Type I interferons	Plasmacytoid DCs	CD8^+^ T cells and NK cells (cytotoxicity)Th1 polarizationB cells (differentiation; IgG class-switching)	?pathogenesis of seropositive disease [16]	Anti-IFNα (sifalimumab)
IL-8 (CXCL8)	Macrophages, epi/endothelial cells.Osteoclasts (?response to autoantibodies)	Neutrophils, leukocytes (chemotaxis)Osteoclasts (activation, including autocrine)	Leukocyte chemotaxis?Bone erosion/pain in ‘pre-RA’ [19^▪▪^]	CXCR1/2 inhibitor (reparixin)

Typical cellular sources and effects in target cells are listed, and broadly accepted roles in rheumatoid arthritis pathogenesis are summarised, along with available/potential therapeutic targeting strategies (exemplar originator agents named).

^a^Only asterisked agents are currently licensed for use in rheumatoid arthritis). Less established roles indicated by ‘?’ GM-CSF, granulocyte macrophage-colony stimulating factor; DC, dendritic cell; ILC, innate lymphoid cell; NK, natural killer; SF, synovial fibroblast.
